# The *Renilla *luciferase gene as a reference gene for normalization of gene expression in transiently transfected cells

**DOI:** 10.1186/1471-2199-11-103

**Published:** 2010-12-31

**Authors:** Meesbah Jiwaji, Rónán Daly, Kshama Pansare, Pauline McLean, Jingli Yang, Walter Kolch, Andrew R Pitt

**Affiliations:** 1 Institute of Molecular, Cell and Systems Biology, College of Medical, Veterinary and Life Sciences, University of Glasgow, Glasgow, G12 8QQ, UK; 2 Inference Group, School of Computing Science, University of Glasgow, Glasgow, G12 8QQ, UK; 3 Systems Biology Ireland and the Conway Institute, University College Dublin, Belfield, Dublin 4, Ireland

## Abstract

**Background:**

The importance of appropriate normalization controls in quantitative real-time polymerase chain reaction (qPCR) experiments has become more apparent as the number of biological studies using this methodology has increased. In developing a system to study gene expression from transiently transfected plasmids, it became clear that normalization using chromosomally encoded genes is not ideal, at it does not take into account the transfection efficiency and the significantly lower expression levels of the plasmids. We have developed and validated a normalization method for qPCR using a co-transfected plasmid.

**Results:**

The best chromosomal gene for normalization in the presence of the transcriptional activators used in this study, cadmium, dexamethasone, forskolin and phorbol-12-myristate 13-acetate was first identified. qPCR data was analyzed using geNorm, Normfinder and BestKeeper. Each software application was found to rank the normalization controls differently with no clear correlation. Including a co-transfected plasmid encoding the *Renilla *luciferase gene (*Rluc*) in this analysis showed that its calculated stability was not as good as the optimised chromosomal genes, most likely as a result of the lower expression levels and transfection variability. Finally, we validated these analyses by testing two chromosomal genes (*B2M *and *ActB*) and a co-transfected gene (*Rluc*) under biological conditions. When analyzing co-transfected plasmids, *Rluc *normalization gave the smallest errors compared to the chromosomal reference genes.

**Conclusions:**

Our data demonstrates that transfected *Rluc *is the most appropriate normalization reference gene for transient transfection qPCR analysis; it significantly reduces the standard deviation within biological experiments as it takes into account the transfection efficiencies and has easily controllable expression levels. This improves reproducibility, data validity and most importantly, enables accurate interpretation of qPCR data.

## Background

The analysis of gene expression using quantitative real-time polymerase chain reaction (qPCR) has become increasingly important as biological research has focused on developing insights into the complex regulatory networks that exist within cells [1]. qPCR is often the assay of choice as it is sensitive and reproducible; it allows the simultaneous analysis of gene expression in a number of different samples and as a result of the high dynamic range, this technique is suitable even when only a few cells are available. The speed of analysis and the potential for automation and multiplexing makes qPCR an attractive technique for the analysis of gene expression [2-4].

Unfortunately, problems attributed to the biological and technical variability which can occur between the different steps of the experimental procedures, are associated with the qPCR assay. The technical variables include the amount of starting materials in the reactions, the quality of the RNA samples and the efficiency of the enzymatic steps (i.e. reverse transcription and PCR) [5,6]. The biological variables include the differences in the levels of transcriptional expression of genes between tissues and cell types [7]. To take into account these variations, internal reference genes are often used to normalize the qPCR data [8,9]. Ideally, the internal reference gene should be expressed at levels comparable to the gene of interest and the levels of expression of the gene selected as the internal reference should not vary between the samples and treatments selected for analysis [8,10]. The selection of the most appropriate internal reference gene serves to decrease the error both within the experiment and between biological experiments [8,9,11]. In addition, it allows valid analyses of qPCR data to be conducted [10]. This makes the selection of the internal reference an important factor in the design of a qPCR experiment. This becomes particularly important when the sequence targeted for analysis is being transiently transfected into cells; however validated methods for this type of experiment are not currently available.

In a living cell, it is unlikely that the transcription of any gene is resistant to changes in the cell cycle or in the levels of nutrients. It is therefore important that in the selection of the reference gene, the candidate genes should be regulated at a minimal level. A number of studies have shown that the classical internal reference genes, such as the glyceraldehyde-3-phosphate dehydrogenase gene (*GAPDH*), are not always the most suitable reference genes [12], and that the levels of GAPDH mRNA fluctuate in the cell. This is understandable considering the many pathways in which this protein is involved, including endocytosis, translational control, export of nuclear tRNA, DNA replication and repair, apoptosis and glycolysis [12]. *GAPDH *was originally selected as a normalization reference as the gene encoded a protein with a 'housekeeping' function. It was not until later that the role of *GAPDH *in the cell was more fully understood, and thus its potential unsuitability as an internal reference. It is, therefore, important that the design of a biological study includes the evaluation of potential internal reference genes, and that the most appropriate reference genes are selected. The availability of software applications such as geNorm, Normfinder and BestKeeper, that use statistical methods to select the most appropriate internal reference genes, make this task easier [13-15].

Most of the traditional internal reference genes are chromosomal genes. The use of a chromosomal internal reference takes into account all the technical and biological variables that are present within the experiment bar one. None of the internal reference genes, as long as the gene was present on the chromosomal DNA, would take into account the variation in the transfection efficiency between samples that had been transiently transfected with plasmid DNA encoding a gene of interest. Also, choosing a genomically encoded gene with an appropriate expression level is challenging. We have adapted a technique often applied to the normalization of reporter enzyme activity to qPCR, and have demonstrated that using a co-transfected plasmid-encoded *Renilla reniformis *luciferase gene (*Rluc*) as an internal reference is more appropriate for the normalization of qPCR data in transiently transfected systems.

## Results and Discussion

We are developing a plasmid vector to study transcriptional activation derived from *cis*-acting regulatory elements of interest in transiently transfected mammalian cell lines. We planned to validate the putative *cis*-acting regulatory elements using a reporter enzyme system that could be adapted to allow a high-throughput analysis of the samples of interest. Traditional reporter enzyme assays usually use the activity of one reporter enzyme to normalize the activity of another enzyme of interest when two plasmids, each separately encoding one of the enzymes, have been transiently transfected into cells. One of the most common systems is the firefly luciferase-*Renilla *luciferase enzyme system, where the enzyme activity of *Renilla *luciferase (encoded by the control plasmid) is used to normalize the firefly luciferase enzyme activity (present on the experimental plasmid).

A complete workflow for the firefly luciferase-*Renilla *luciferase system from the plasmid constructs used to transfect the cells to the reporter enzyme assay system used to quantify the reporter enzyme activity has been commercially developed (Promega). This uses one of three control plasmid constructs with different promoters upstream of the *Rluc *gene [16]. pRL-SV40, which encodes the early simian virus 40 (SV40) enhancer/promoter region, and pRL-CMV, which encodes the cytomegalovirus (CMV) immediate early enhancer/promoter region both provide high levels of *Renilla *luciferase enzyme expression. In contrast, pRL-TK encodes the weaker herpes simplex virus (HSV) thymidine kinase (TK) promoter, resulting in lower levels of *Renilla *luciferase enzyme expression.

For the studies described here the most appropriate system would have a promoter upstream of *Renilla *luciferase with relatively high expression levels such that a low concentration of the control plasmid could be used in the transient transfections. It would also be advantageous to use a promoter that did not encode transcription factor binding sequences. All three control plasmids provided as part of the commercial kit have their limitations. Breuning et al. [17] and Svensson et al. [18] reported that the CMV promoter was susceptible to up-regulation in mammalian cells under conditions of cell stress. This is not ideal since in *cis*-regulatory element studies it is likely that some of the treatments used to activate the *cis*-regulatory elements will result in cell stress. A number of reports have also highlighted that HSV TK promoter is susceptible to altered regulation under a range of experimental conditions [19-21]. Ho et al. [21] performed a comparison between pRL-TK and pRL-SV40 and stated that of the two plasmids, pRL-SV40 was less susceptible to non-specific activation by the GATA transcription factor than pRL-TK. As this project aims to study *cis*-acting regulatory elements, we wanted to select the control plasmid that was least affected by undesired transcriptional regulation so we selected pRL-SV40 as the control plasmid.

**Figure 1:**
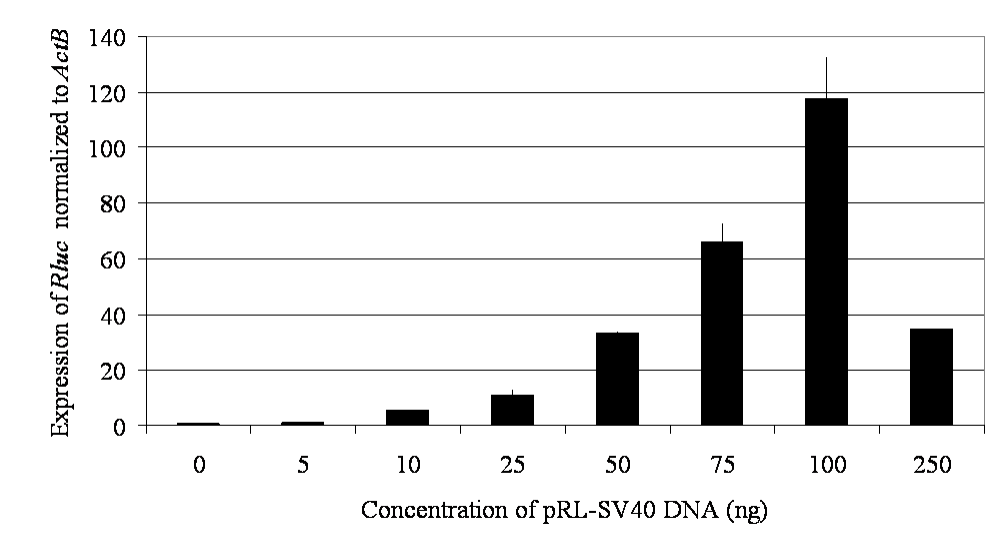
**The effect of an increasing transfection concentration of pRL-SV40 DNA, a control plasmid encoding *Rluc*, on *Rluc *gene expression**. *Rluc *expression was normalized to *ActB *gene expression. Each sample represents the expression of *Rluc*/*ActB *in a total RNA concentration of 0.2 μg.

To test pRL-SV40 (the control plasmid encoding *Rluc*), human embryonic kidney (HEK) 293 cells were transiently tranfected with an increasing concentration of pRL-SV40. Using a qPCR assay, expression of *Rluc *was normalized to the expression of *ActB*, a chromosomal gene that is often used as a reference control [12]. As the DNA concentration of pRL-SV40 used to transfect the cells increased, increasing *Rluc *expression was detected (Figure 1) and optimal *Rluc *expression was detected when HEK293 cells were transfected with 100 ng of control plasmid DNA under the conditions being used. In these experiments, we needed to balance the concentration of the control plasmid used in the transfections with the level of *Rluc *expression. The lowest concentration of control plasmid DNA at which consistent levels of *Rluc *gene expression were obtained was with 50 ng of pRL-SV0 so this concentration of control plasmid DNA was used in subsequent experiments. It is interesting to note that the use of a transfected gene for normalization is advantageous in that the levels of the transfected gene can easily be adjusted to be suitable for a broad range of target genes by altering the absolute amount of plasmid DNA in the transfection.

HEK293 cells were transiently tranfected with pRL-SV40 (50 ng) and an equivalent concentration of our reporter system plasmids pMN85 or pMN86. The concentration of pMN85/pMN86 was determined in an experiment similar to the one that was conducted for pRL-SV40 (data not shown). The control plasmid, pRL-SV40, is 3.7 kb in size compared to pMN85/pMN86, both of which are 5.1 kb in size. Yin et al. [22] reported that there was an inverse correlation between the plasmid size and the transfection efficiency of that plasmid. As both the reporter system plasmids are of comparable size (pMN86 is 82 bp larger than pMN85), we determined that the transfection efficiencies between these plasmids would not be significantly different. In addition, despite the differences in the transfection efficiencies of the reporter system plasmids and the control plasmid, the transfection efficiencies of the respective plasmids should be comparable between samples allowing us to normalize the data derived from the reporter system plasmids to that of pRL-SV40, the control plasmid.

**Figure 2:**
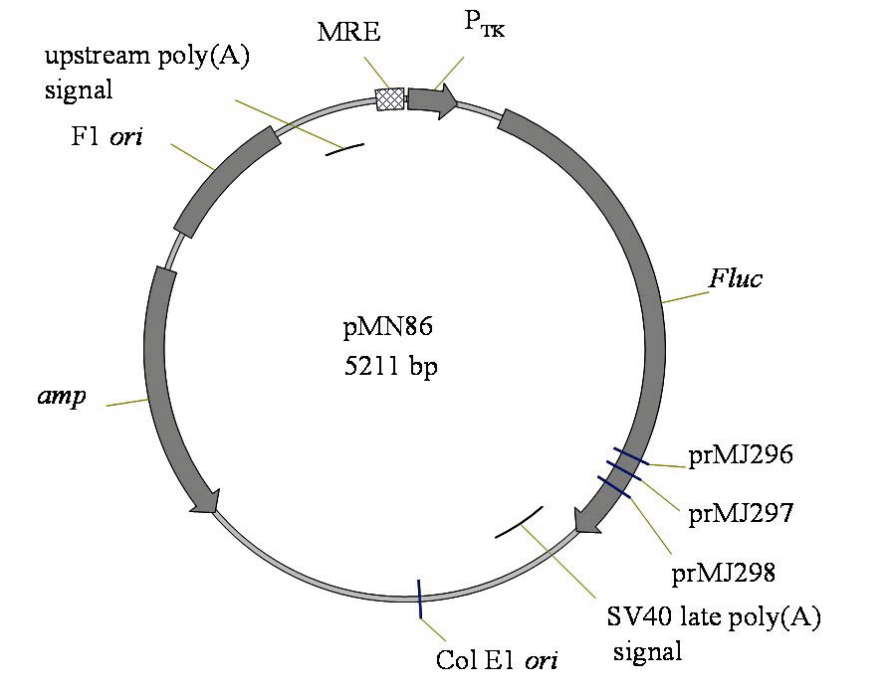
**Schematic map of the plasmid pMN86**. The plasmid encodes origins of replication (Col E1, FI *ori*), a gene conferring resistance to the antibiotic ampicillin (*amp*), the thymidine kinase promoter (P_TK_) upstream of the Firefly luciferase gene (*Fluc*) and five copies of the Cd-responsive element (MRE). The MRE- P_TK _*-Fluc *cassette is flanked by two poly(A) signals to prevent transcriptional interference.

Plasmid pMN85 is the control vector which encodes the thymidine kinase promoter (P_TK_) upstream of the firefly (*Photinus pyralis*) luciferase gene (*Fluc*). In pMN86, a putative metal responsive element (MRE) is also present upstream of the P_TK _(Figure 2). Using the dual luciferase reporter assay system (Promega), we confirmed that the putative MRE conferred high cadmium (Cd) inducibility to the P_TK _in transiently transfected HEK293 cells (Table 1; pMN86 (MRE-P_TK_*-Fluc*) vs. pMN85 (P_TK_*-Fluc*)).

**Table 1:** Cd-dependent induction of MRE-directed expression of the firefly luciferase reporter enzyme activity

	Normalized Fold Induction
pMN85 (P_TK_*-Fluc*)	1.00 ± 0.00
pMN86 (MRE-P_TK_*-Fluc*)	212.77 ± 8.92

The firefly and *Renilla *luciferase enzyme assays were conducted using the dual luciferase reporter assay system (Promega). The firefly luciferase enzyme activity was normalized to *Renilla *luciferase enzyme activity

In order to quantify the transcriptional activity of the induction of the Cd responsive MRE sequence using qPCR, we needed to find an appropriate normalization reference for these experiments. We planned to test the induction of *cis*-acting regulatory elements in the presence of a variety of compounds including metals (i.e. Cd), steroids (i.e. dexamethasone), cAMP activators (i.e. forskolin) and activators of protein kinase C (i.e. phorbol-12-myristate 13-acetate (TPA)). Hence, the reference gene selected as a normalization control had to be stably expressed and unaffected by these inducers.

Traditionally, a chromosomal gene is selected as a reference gene for normalization. To this end, we tested a panel of 11 chromosomal genes as internal controls for qPCR normalization using polymerase chain reaction (PCR) primers and fluorescent probes provided in the geNorm Housekeeping Selection Kit (PrimerDesign Ltd). The panel included the genes for 18S rRNA (*18S*), beta-actin (*ActB*), ATP synthase (*Atp5B*), beta-2-microglobulin (*B2M*), cytochrome c-1 (*Cyc1*), isoform 2 of the eukaryotic translation initiation factor 4A (*Eif4A2*), *GAPDH*, ribosomal protein L13a (*RPL13A*), subunit A of the succinate dehydrogenase complex (*SDHA*), DNA topoisomerase I (*TOP1*) and the zeta polypeptide of the tyrosine-3-monooxygenase/tryptophan-5-monooxygenase activation protein (*YWHAZ*). The expression of these 11 chromosomal genes was measured in HEK293 cells that had been co-transfected with pMN85 or pMN86 and pRL-SV40 in the absence and presence of Cd, dexamethasone, forskolin and TPA. The data was compared using three software applications; geNorm, Normfinder and BestKeeper (websites are shown in the Methods section).

GeNorm, a Visual Basic Application for Microsoft Excel, determines the expression stability of a potential reference gene by assigning each gene in a set of candidate reference genes an M value [13]. This M value is the mean pair-wise variation between any two genes in the data set being analyzed, one designated as the candidate gene and the other as the candidate reference gene. The gene with the highest M value is then removed, and the stability measure of the remaining genes recalculated. As the calculation of M value requires two genes, the calculation proceeds until the two best normalization reference genes, with the lowest M value, remain in the analysis. As the expression profiles of the genes in the data set are compared to each other, the quality of the 'best' reference gene is dependent on the other genes that are selected for analysis, such that the geNorm ranking is reliable only if most of the genes selected as candidate reference genes are stably expressed and that the candidate reference genes are not co-regulated. To analyze our data with geNorm, the quantification cycle (Cq) values generated in the qPCR experiments were converted to relative levels of expression using the method described by Livak and Schmittgen [23] and Vandesompele et al. [13], such that the lowest Cq value was set to 1 and all the other levels of expression for that gene were related to the lowest Cq value by the delta Ct method. These delta Cq values were then used to calculate the M values. geNorm analysis of the qPCR data for the 11 chromosomal reference genes (Table 2), indicated that the least suitable chromosomal reference gene was *SDHA *(M = 2.77) and the most stable were *B2M *and *YWHAZ *(M = 0.10).

**Table 2:** Ranking of chromosomal reference genes based on geNorm, Normfinder and BestKeeper analyses

	geNorm		Normfinder			BestKeeper		
	M value	Ranking	Stability value	Standard error	Ranking	[r]	p-value	Ranking
*18S*	0.13	3	1.425	1.540	8	0.958	0.003	6
*ActB*	0.30	10	0.344	2.113	4	0.914	0.011	8
*Atp5B*	0.22	7	0.255	2.539	3	0.973	0.001	4
*B2M*	**0.10**	**1/2**	1.449	1.541	9	**0.974**	**0.001**	**1/2/3**
*Cyc1*	0.16	4	1.126	1.543	6	0.933	0.007	7
*Eif4A2*	0.24	8	0.204	2.981	2	**0.974**	**0.001**	**1/2/3**
*GAPDH*	0.20	6	0.444	1.872	5	0.881	0.020	9
*RPL13A*	0.19	5	**0.195**	**3.090**	**1**	**0.974**	**0.001**	**1/2/3**
*SDHA*	2.77	11	31.894	10.087	11	nd	nd	nd
*TOP1*	0.27	9	2.002	1.580	10	0.704	0.119	10
*YWHAZ*	**0.10**	**1/2**	1.225	1.539	7	0.959	0.002	5

Highlighted regions represent the best ranked gene in the analysis. Abbreviations: nd- not determined; M value- geNorm measure of expression stability, [r]- BestKeeper coefficient of correlation, p-value- BestKeeper probability value

Normfinder is also a Visual Basic Application and it too assigns a stability value to the candidate reference genes. In contrast to geNorm, Normfinder uses a model-based approach to provide a value for the two most stable reference genes with the least intra- and inter-group variation [15]. The robustness of the Normfinder approach for the selection of reference genes for normalization was demonstrated by Andersen et al. [15]. As for the geNorm analysis, the experimental Cq values were converted to a linear scale of expression using the delta Ct method. This data loaded into Normfinder in two ways- (1) with the data separated into groups corresponding to treatment with the different activators, and (2) with all the treatment groups combined. In Normfinder, stability is expressed as a stability value in arbitrary units. Analysis of our data (Table 2) indicated that the *RPL13A *chromosomal gene was the most stable (stability value = 0.195 ± 3.090) and that again *SDHA *was the least stable gene (stability value = 31.894 ± 10.087).

BestKeeper is an Excel-based tool that calculates the variability in the expression of the pool of reference genes by analyzing the Cq values directly and defining variability by the standard deviation (SD) and coefficient of variance (CV) [14]. All genes with SD values of greater than 1 are considered to be unsuitable reference genes. The gene with the lowest SD is considered to be the most suitable reference gene. BestKeeper then performs a comparative analysis based on the pair-wise correlations of the all the candidate reference genes to each other and generates a BestKeeper index. The comparison of this BestKeeper index to each candidate reference gene, using pair-wise correlation analyses, results in each candidate reference gene being assigned a value for the Pearson correlation coefficient (r) and the probability (p). The most suitable reference gene is that with an r value closest to 1.0, with the p value providing an indication of the significance of the r value. The most unstable gene in the two previous analyses, *SDHA*, was not included in the BestKeeper analysis. The BestKeeper analysis (Table 2) of the potential reference genes highlighted *B2M*, *Eif4A2 *and *RPL13A *as being the most suitable normalization reference genes (r = 0.974, p = 0.001) and *TOP1 *as the least suitable (r = 0.704, p = 0.119).

When the results of the geNorm, Normfinder and BestKeeper analyses were summarized, the most stable reference genes were *B2M *and *RPL13A*, as they were ranked as the most stable genes by two of the three software applications (Table 2). *ActB *is a chromosomal gene that is often used as a reference control. In fact, Suzuki et al. [12] report that *ActB *was one of the most popular reference controls, second only to *GAPDH*, being used in 32% of the cases examined. For these reasons, we selected *B2M *and *ActB *as reference controls in further analyses. To compare *Rluc *on a transfected plasmid to the selected chromosomal reference genes (*B2M *and *ActB*) directly, the experimental Cq data for *Rluc *was incorporated into the geNorm, Normfinder and BestKeeper analysis (Table 3).

**Table 3:** Comparison of the ranking of B2M and ActB, the selected chromosomal reference genes, and Rluc, present on a transiently co-transfected plasmid, based on geNorm, Normfinder and BestKeeper analyses

	geNorm		Normfinder			BestKeeper		
	M value	Ranking	Stability value	Standard error	Ranking	[r]	p-value	Ranking
*ActB*	**0.44**	**1/2**	1.342	0.515	3	0.931	0.001	2
*B2M*	**0.44**	**1/2**	**0.432**	**0.955**	**1**	**0.985**	**0.007**	**1**
*Rluc*	0.63	3	1.236	0.507	2	0.694	0.001	3

Highlighted regions represent the best ranked gene in the analysis. Abbreviations: nd- not determined; M value- geNorm measure of expression stability, [r]- BestKeeper coefficient of correlation, p-value- BestKeeper probability value

The stability values for *Rluc *were generally lower than for the chromosomal genes (0.63 (geNorm), 1.236 (Normfinder) and 0.694 (BestKeeper), as might be expected, since this reflects both the problem of variable transfection efficiency, and the lower levels of expression, meaning that more rounds of PCR are needed, accentuating any variable transfection efficiencies. However, the scores were still better than some, and close to the majority, of the generally used chromosomal genes, suggesting that this could still be considered as a candidate system for normalization [24-26]. According to the geNorm manual [http://medgen.ugent.be/~jvdesomp/genorm/geNorm_manual.pdf], genes with M≥1.5 are deemed unsuitable as reference genes. Based on this cut-off point, *Rluc *is not excluded as a suitable reference gene. Unfortunately, neither Andersen et al. [15] nor Pfaffl et al. [14] have reported guidelines on the range of acceptable stability values for the output of the Normfinder or BestKeeper applications, respectively [14,15]. This makes it difficult to determine the suitability of *Rluc *as a normalization control based on the values of the output of the Normfinder and BestKeeper applications. The output of all three bioinformatic analyses indicated that *Rluc *was not the most suitable gene for use as a reference control.

To evaluate the data from the geNorm, Normfinder and BestKeeper analyses for its biological validity, *Rluc *was compared to two chromosomal genes (*B2M *and *ActB*) for its expression stability as a reference gene for normalization of a transfected plasmid expressing *Fluc*. qPCR was conducted on cDNA samples generated from HEK293 cells transfected with pMN85 (P_TK_*-Fluc*) or pMN86 (MRE-P_TK_*-Fluc*) and pRL-SV40 (*Rluc*). The Cq values obtained for the chromosomal internal controls *B2M *and *ActB *were between 19 and 20 cycles, which were significantly lower than the Cq obtained for *Rluc *and for the target sequence, *Fluc*, which showed average Cq values of between 27 and 28 cycles (Table 4).

**Table 4:** Cq values obtained for Fluc, Rluc, B2M and ActB in qPCR reactions

	Gene			
	*Fluc*	*Rluc*	*B2M*	*ActB*
Cq value ± SD	27.12 ± 2.76	27.98 ± 0.44	19.92 ± 0.42	19.08 ± 0.52

Abbreviations: Cq- quantification cycle, SD- standard deviation

**Figure 3:**
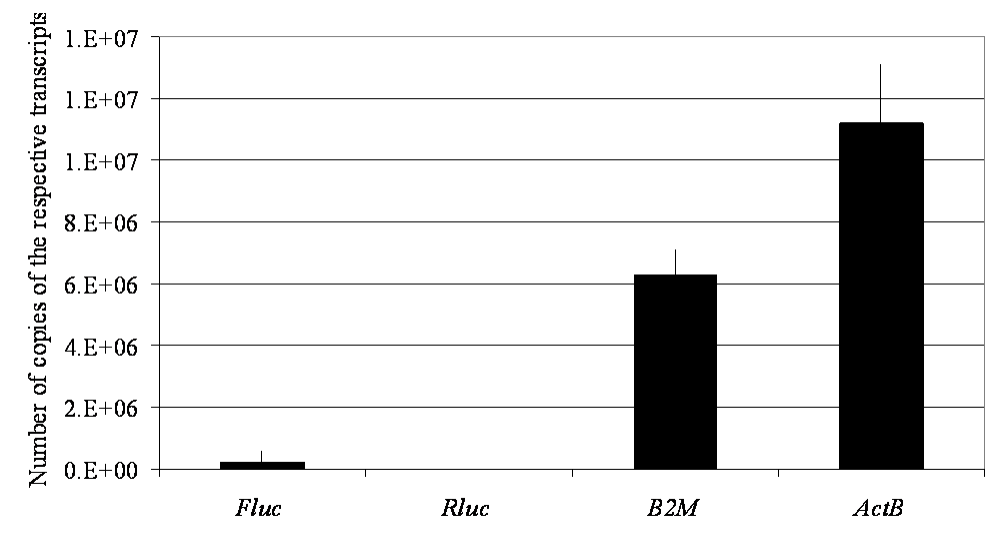
**Average number of copies of the *Fluc, Rluc, B2M and ActB *transcripts**. HEK293 cells were transiently transfected with pMN85/pMN86 and pRL-SV40. The average numbers of copies represent those present in mRNA isolated from 0.2 μg of total RNA.

The Cq values were converted to number of copies with standard curves generated using known DNA concentrations of the respective linear DNA that encoded *Fluc*, *Rluc*, *B2M *or *ActB *in qPCR reactions. The average numbers of copies of *B2M *and *ActB *in the cDNA reactions in this experiment were 1500-fold and 2700-fold higher than the average number of copies of *Rluc *(Figure 3). In contrast, the average number of copies of *Fluc *(under both control and induced conditions) was 30 fold higher than the average number of copies of *Rluc*. This highlighted the first problem associated with the selection of a chromosomal gene as an internal reference for the normalization of qPCR data from cells that had been transiently transfected with sequences of interest. The levels of expression of the chromosomal genes were much higher than the levels of the genes that were transiently transfected into cells. Problems associated with using highly expressed chromosomal genes as reference controls have been noted before, for example by Frost and Nilsen [27]. In addition, it has been noted that the use of reference and target genes with highly variable levels of expression would increase the noise within the assay system potentially resulting in a reduced sensitivity [8,10]. These dissimilar levels of expression were made more apparent when the number of copies of *Fluc *was normalized to the internal reference genes as the ratios for normalized copies for *Fluc/B2M *and *Fluc/ActB *were very small compared to the ratio of *Fluc/Rluc *(Table 5).

**Table 5:** Copies of Fluc normalized to the copies of the reference genes Rluc, B2M and ActB

		*Fluc/Rluc*		*Fluc/B2M*		*Fluc/ActB*	
		Normalized copies	± SD	**Normalized copies (× 10**^**-3**^**)**	± SD	**Normalized copies (× 10**^**-3**^**)**	± SD
pMN85	Untreated	3.91	0.53	2.73	0.34	1.41	0.23
	+ Cd	5.11	1.00	2.85	0.46	1.86	0.25
pMN86	Untreated	3.50	0.51	2.22	0.43	1.22	0.31
	+ Cd	211.35	19.46	155.77	29.88	86.30	12.42

The number of copies of the genes (*Fluc*/*Rluc*/*B2M*/*ActB*) was calculated using standard curves generated as described in the Methods. The normalized copy numbers for *Fluc/B2M *and *Fluc/ActB *were multiplied by a factor of (1 × 10^3^) to allow the comparison of the ratio of *Fluc *normalized to the chromosomal reference genes (*B2M *and *ActB*) to the ratio of *Fluc *normalized to the transiently co-transfected gene (*Rluc*). Abbreviations: SD- standard deviation

The system was then tested for measuring changes in transcription levels. Following induction of the MRE-directed transcription of *Fluc *in the presence of Cd and normalization against the internal chromosomal reference genes *B2M *and *ActB*, a 71-fold and 75-fold change in *Fluc *expression was calculated respectively (Table 6). In contrast, when *Fluc *was normalized to co-transfected *Rluc*, a lower 61-fold induction was calculated (Table 6). While the levels of fold induction of the Cd-responsive promoter-directed transcription of *Fluc *were not statistically different regardless of which gene was used as a normalization control, the levels of fold induction using the chromosomal genes as standards were consistently higher than when *Rluc *was used as the normalization control. More importantly, when the *Fluc *was normalized to *B2M *and *ActB*, the error in the measurement, expressed as standard deviation, increased considerably to 23% and 35% of the total fold induction respectively compared to normalization to *Rluc*, where the standard deviation was 16% (Table 6). This is supported by the statistical analysis of the data which demonstrated that the calculated intervals for the fold induction and the standard deviation were smaller and less variable when *Fluc *was normalized to *Rluc *as opposed to *B2M *or *ActB *(Table 7). This dramatic decrease in the standard deviation is most likely because, in addition to accounting for the quantity of RNA present in the starting material, the quality of the RNA and the efficiency of the enzymatic reactions during the experimental process, normalization of *Fluc *to *Rluc *also took into account the differences in the transfection efficiencies within the experiment.

**Table 6:** Induction of Fluc expression in the presence of Cd

	Fold induction		
	*Fluc/Rluc *± SD (% SD)	*Fluc/B2M *± SD (% SD)	*Fluc/ActB *± SD (% SD)
pMN85	1.34 ± 0.42	1.07 ± 0.27	1.35 ± 0.28
pMN86	61.42 ± 10.12 (16%)	71.38 ± 16.24 (23%)	74.92 ± 25.80 (35%)

The normalized copy number of the Cd-treated samples was compared to the normalized copy number of the untreated samples for pMN85 (P_TK_*-Fluc*) and pMN86 (MRE-P_TK_*-Fluc*) providing values for the fold induction of the Cd-directed *Fluc *expression for each of the reference genes (*Rluc*, *B2M *and *ActB*). Abbreviations: SD- standard deviation

**Table 7:** Credible Intervals for Fluc expression in the presence of Cd

	95% Credible intervals for fold induction		
	*Fluc/Rluc*	*Fluc/B2M*	*Fluc/ActB*
Mean fold induction	(53.11,77.52)	(58.92, 106.48)	(59.98, 121.00)
Standard deviation	(9.19, 34.85)	(16.30, 74.62)	(18.60, 105.60)

The data were modelled using a lognormal distribution and uninformative priors. Random samples from the posterior were drawn and 95% credible intervals were calculated for both the mean fold induction and standard deviation.

## Conclusions

A good control for normalization in qPCR experiments should be stably expressed at levels similar to those of the genes of interest, and should take into account all factors that could affect expression levels in the experiment, including transfection efficiency. While we observed that the chromosomal internal control genes *B2M *and *ActB *were more stably expressed than transfected *Rluc *under the experimental conditions, the levels of expression of *B2M *and *ActB *were much higher than those of genes transiently transfected into the cell. The *Rluc *gene was expressed at levels similar to the gene of interest and was demonstrated by a number of biological analyses to be the most appropriate control, ensuring that differences in the transfection efficiencies between the samples were taken into account, thus significantly improving the reproducibility and the validity of the qPCR experiments.

## Methods

### General Culture Conditions

*Escherichia coli *DH5α cells [28] were used for the construction, screening and propagation of plasmid constructs. *E. coli *DH5α cells were cultured in lysogeny broth (LB) or on LB agar containing 100 μg/mL ampicillin as described by Sezonov et al. [29]. HEK293 cells (LGC Standards, Middlesex, UK; ATCC Number CRL-1573) [30] were maintained in Dulbecco's Modified Eagle Medium (DMEM, Invitrogen Ltd, Paisley, UK; 41966-029) supplemented with 4 mM L-glutamine (Sigma-Aldrich, Dorset, UK; G7513) and 10% foetal bovine serum (FBS; Biosera, East Sussex, UK; S1810/500) at 37°C in an atmosphere that contained 5% CO_2 _as per the supplier's propagation instructions.

### Chromosomal reference genes

The geNorm Housekeeping Gene Selection Kit (PrimerDesign Ltd, Southampton, UK; ge-PP-12-hu) was used as the panel of chromosomal reference genes. It included forward primers, Fluorescein (FAM)-labelled fluorescent probes and reverse primers for the chromosomal genes for *18S*, *ActB*, *Atp5B*, *B2M*, *Cyc1*, *Eif4A2*, *GAPDH*, *RPL13A*, *SDHA*, *TOP1 *and *YWHAZ*.

### Description of plasmids pMN85 and pMN86

Plasmid pMN85 was derived from pGL3 Basic (Promega Corporation, Madison, WI, USA; E1751). DNA encoding (1) a multiple cloning cassette into which transcription factor binding sites could be inserted for analysis and (2) a P_TK _was inserted between the *Kpn *I and *Hind *III restriction enzyme sites upstream of *Fluc *in pGL3 generating pMN85. In pMN86 (Figure 2), five copies of a nucleotide sequence sensitive to induction in the presence of Cd (MRE; CGG GTG CGC CCG GCC CGA) were inserted between the *Eco *RI and *Eco *RV restriction enzyme sites upstream of P_TK_.

### Transfection of HEK293 cells

Plasmid DNA was prepared using the PureYield Plasmid Midiprep System (Promega Corporation, Madison, WI, USA; A2495). 1 × 10^6 ^HEK293 cells and, unless otherwise stated, a total of 100 ng DNA (50 ng pRL-SV40 (Promega Corporation, Madison, WI, USA; E2231) and 50 ng of either pMN85 or pMN86) was used in each transfection. HEK293 cells were transfected using Genejuice (Novagen supplied by Merck Chemicals Ltd, Nottingham, UK; 70967-6) as recommended by the manufacturers instructions. Transfected HEK293 cells were incubated for 24 hours and subsequently induced with 12.5 μM CdCl_2 _(Sigma-Aldrich, Dorset, UK; 439800), 2.5 μM dexamethasone (Sigma-Aldrich, Dorset, UK; D8893), 25 μM forskolin (Sigma-Aldrich, Dorset, UK; F6886) or 0.25 μM TPA (Sigma-Aldrich, Dorset, UK; P1585) for 4 hours. Each experiment contained n = 6 biological replicates and experiments were repeated at least three times. Results between the experiments were comparable.

### Reporter enzyme assays

The firefly and *Renilla *luciferase activities in 20 μL HEK293 cell lysate were quantified with the dual luciferase reporter assay system as per the manufacturer's instructions (Promega Corporation, Madison, WI, USA; E1910) in a Luminoskan Ascent (Thermo Fisher Scientific, Waltham, Ma, USA) luminometer. The firefly and *Renilla *luciferase enzyme activities were measured for each biological sample. The firefly luciferase enzyme activity was normalized to the *Renilla *luciferase enzyme activity. The Cd-treated samples were then normalized to untreated samples yielding values for the fold induction of the firefly luciferase enzyme activity directed by the MRE in the presence of Cd.

### RNA purification and cDNA synthesis

Total RNA was prepared from 1 × 10^6 ^HEK293 cells using the miRNeasy mini kit (Qiagen Ltd, West Sussex, UK; 217004), mRNA was isolated from 4 μg of total RNA with 25 μL of the Dynabeads mRNA Purification Kit (Invitrogen Ltd, Paisley, UK; 610-06) and cDNA produced from the purified mRNA with 100 ng random hexamers (Invitrogen Ltd, Paisley, UK; 48190011) or 500 μg/mL oligo dT_(12-18) _primer (Invitrogen Ltd, Paisley, UK; 18418-012), 2.5 mM of each dNTP (Promega Corporation, Madison, WI, USA; U1240), 40 U RNaseOUT (Invitrogen Ltd, Paisley, UK; 10777-019), and 100 U Superscript II enzyme (Invitrogen Ltd, Paisley, UK; 18064014) in a final volume of 20 μL. The cDNA synthesis reaction was incubated at 25°C for 10 minutes, 42°C for 60 minutes and finally, at 70°C for 15 minutes.

### qPCR conditions

The cDNA (20 μL) was diluted to 80 μL with RNase-free H_2_0 and 4 μL was used in each qPCR reaction. For qPCR analysis of the chromosomal reference genes, in addition to 4 μL of cDNA, 300 nM proprietary primer mixture (PrimerDesign Ltd, Southampton, UK; ge-PP-12-hu), which includes the forward primer, the FAM-labelled fluorescent probe and the reverse primer, and 1 × Lightcycler Probes Master (Roche Diagnostics Ltd, Burgess Hill, UK; 04887301001) was used. For the qPCR analysis of *Fluc*, *Rluc*, *B2M *and *ActB *gene expression, in addition to the cDNA, each reaction contained 2.4 μM forward primer, 2.4 μM reverse primer, 0.15 μM fluorescent probe and 1 × Lightcycler Probes Master. All primers were designed with Primer3Plus http://www.bioinformatics.nl/cgi-bin/primer3plus/primer3plus.cgi, synthesized by Eurofins MWG (Ebersberg, Germany) and the nucleotide sequence of the primers used in each reaction are shown in Table 8. The cDNA was analyzed in a Lightcycler 480 (Roche Diagnostics Ltd, Burgess Hill, UK) using the following amplification conditions: (1) denaturation at 95°C for 10 minutes, (2) 50 cycles of amplification at 95°C for 15 seconds then 60°C for 30 seconds after which the level of fluorescence in the sample was measured and finally, (3) cooling to 40°C for 30 seconds.

**Table 8:** Nucleotide sequences of the primers used in the qPCR reactions and the length of the amplicons generated

Transcript (amplicon length)	Primer	Sequence (5' to 3')
*Fluc *(137 nts)	prMJ296	AGGTGGCTCCCGCTGAAT
	prMJ297	FAM-CGGGAAGACCTGCGACACCTGCGT-BHQ1
	prMJ298	CATCGTCTTTCCGTGCTCCA

*Rluc *(140 nts)	prMJ274	GCAGAAGTTGGTCGTGAGG
	prMJ272	HEX-CTCACTATAGGCTAGCCACCATGACTTCGAAAG-BHQ1
	prMJ276	TCATCCGTTTCCTTTGTTCTG

*B2M *(125 nts)	prMJ348	TCTCTGCTCCCCACCTCTAA
	prMJ349	FAM-CCAGCCCTCCTAGAGCTACC-BHQ1
	prMJ350	ATCTGAGCAGGTTGCTCCAC

*ActB *(142 nts)	prMJ351	CTCGGCCACATTGTGAACTT
	prMJ352	FAM-ATGCTCGCTCCAACCGAC-BHQ1
	prMJ353	AACGGTGAAGGTGACAGCA

Abbreviations: BHQ1- Black Hole Quencher 1, FAM- Fluorescein, HEX- hexachlorofluorescein phosphoramidite

### Analysis of the qPCR data for the reference genes

Cq values obtained for HEK293 cells transiently transfected with pMN85/pMN86 and pRL-SV40 were grouped into 5 sets: (1) Untreated samples, (2) + Cd, (3) + dexamethasone, (4) + forskolin and (5) + TPA. Each subset contained n = 6 biological replicates. These sets of data were analyzed with three applications- geNorm http://medgen.ugent.be/~jvdesomp/genorm/, Normfinder http://www.mdl.dk/publicationsnormfinder.htm and BestKeeper http://www.gene-quantification.com/bestkeeper.html#download as described by the documentation for the software applications.

### Generation of standard curves for *Fluc, Rluc, B2M *and *ActB*

Linear dsDNA was used to generate standard curves for qPCR, after Hou et al. [31] highlighted the benefits of using linear DNA in preference to circular DNA. The *Fluc*, *Rluc*, *B2M *and *ActB *PCR products generated in the qPCR reactions above were cloned into the pGEM-T-Easy plasmid using the pGEM-T-Easy Vector System I (Promega Corporation, Madison, WI, USA; A1360). Ligations were transformed into Subcloning Efficiency™ DH5α™ Competent Cells (Invitrogen Ltd, Paisley, UK; 18265-017). Plasmid constructs were screened for the desired insert using the GoTaq^® ^Flexi DNA Polymerase (Promega Corporation, Madison, WI, USA; M8291) in PCR reactions that contained 2 mM MgCl_2_, 25 ng DNA, 0.2 mM of each dNTP (Promega Corporation, Madison, WI, USA; U1240), 1 μM gene specific forward primer (Table 8), 1 μM gene specific reverse primer (Table 8) and 0.5 U GoTaq DNA polymerase in a final volume of 25 μL. The PCR conditions were: (1) 92°C for 2 minutes, 1 cycle; (2) 92°C for 30 seconds, 52°C for 30 seconds, 72°C for 1 minute, 30 cycles and (3) 72°C for 2 minutes, 1 cycle. The DNA in the PCR reactions was separated on a 1% Seakem LE Agarose gel (Lonza Biologics plc, Slough, Berkshire, UK; 50004) containing 0.01% Sybr Safe DNA gel stain (Invitrogen Ltd, Paisley, UK; S33102). Constructs that showed a positive PCR reaction were selected for validation by DNA sequencing.

The PCR fragment of interest (*Fluc*/*Rluc*/*B2M*/*ActB*) was amplified from the pGEM-T-Easy plasmid construct encoding the DNA of interest using the reagents and reaction conditions shown for the qPCR reactions. The PCR products were purified from a 1% Seakem LE Agarose gel (Lonza Biologics plc, Slough, Berkshire, UK; 50004) containing 0.01% Sybr Safe DNA gel stain (Invitrogen Ltd, Paisley, UK; S33102) using the Zymoclean™ Gel DNA Recovery Kit (Zymo Research Corporation, Orange, CA, USA; D4007) and quantified on a Nanodrop spectrophotometer (Thermo Fisher Scientific, Waltham, Ma, USA). The DNA copy number was calculated based on the formulas reported by Whelan et al. [32].

$$\begin{array}{l} \text{Weight in Daltons (g/mol)}=(\text{bp size of ds product})\text{ (33}0\text{ Da}\times \text{2 nt/bp)}\\ \text{Copy number}=\text{Weight in Daltons (g/mol)/Avogadro's number (6}.0\text{22}\times \text{1}0^{\text{23}}\text{ 1/mol)} \end{array}$$

where bp- base pairs, ds- double stranded and nt- nucleotides. With the DNA concentration and the copy number, an accurate number of molecules could be calculated. The DNA was diluted from 1 × 10^9 ^copies/uL to 0 copies/uL in a 10-fold dilution series. qPCR reactions were conducted on the DNA dilutions using the same reagents and reaction conditions reported for the qPCR reactions above. A standard curve was generated by plotting the Cq value against the log of the copy number. The unknown samples were compared to the standard curve and the copy number of the unknown targets calculated.

$$\text{E}=10^{\left(-\text{1}/\text{slope of the curve}\right)}$$

where the optimal efficiency of the qPCR reaction was 2. Primer pairs that have qPCR efficiencies of between 1.6 and 2.4 are typically used [33]. The efficiency of the qPCR reactions and errors were calculated with the Lightcycler 480 qPCR software (Roche Diagnostics Ltd, Burgess Hill, UK) and are shown in Table 9.

**Table 9:** The efficiencies and errors of the qPCR standard curves as calculated by the Lightcycler 480 software (Roche Diagnostics Ltd, Burgess Hill, UK)

Standard curve	Efficiency	Error
*Fluc*	1.878	0.001
*Rluc*	1.878	0.001
*B2M*	2.027	0.053
*ActB*	1.994	0.002

### qPCR data analysis

Cq values obtained for HEK293 cells transiently transfected with pMN85/pMN86 and pRL-SV40 were grouped into 4 sets: (1) pMN85 - Untreated, (2) pMN85 - + Cd, (3) pMN86 - Untreated, (4) pMN86 - + Cd. The Cq values were converted to copy numbers using the *Fluc*, *Rluc*, *B2M *and *ActB *standard curves. The copy number of *Fluc *was normalized to the copy number of *Rluc*, *B2M *or *ActB*. The copy number of the normalized Cd-treated samples were then compared to the copy number of the normalized untreated samples for pMN85 and pMN86 yielding values for the fold induction of the *Fluc *directed by the MRE in the presence of Cd.

### Statistical analysis of qPCR data

The data were analysed using JAGS [34], a Gibbs sampler for hierarchical models and Coda [35], a tool for examining Markov Chain Monte Carlo runs. The data were modelled using a log-normal distribution, with uninformative normal priors for the mean parameter and uninformative gamma priors for the precision. For each set of data, 10 independent runs were used, each with a burn in of 20000 iterations and 100000 samples taken. From these samples, 95% credible intervals were calculated for the posterior mean and standard deviation of the fold induction (Table 7).

## List of Abbreviations

*18S*: 18S rRNA gene; *ActB*: beta actin gene; *Atp5B*: ATP synthase gene; *amp*: β-lactamase gene conferring resistance to ampicillin; *B2M*: beta-2-microglobulin gene; BHQ1: Black Hole Quencher 1; bp: base pairs; Cd: Cadmium; CMV: cytomegalovirus; Cq: quantification cycle; Ct: threshold cycle; CV: coefficient of variance; DMEM: Dulbecco's Modified Eagle Medium; ds: double stranded; E: efficiency of the qPCR reaction; *Eif4A2*: eukaryotic translation initiation factor 4A, isoform 2 gene;FAM:Fluorescein;FBS:foetal bovine serum; *Fluc*: firefly luciferase gene; *GAPDH*: glyceraldehyde-3-phosphate dehydrogenase gene; HEK293: Human Embryonic Kidney 293; HEX: hexachlorofluorescein phosphoramidite; *HMBS*: hydroxymethylbilane synthase gene; HSV: herpes simplex virus; LB: lysogeny broth; M value: geNorm measure of expression stability; MRE: metal responsive element;nt:nucleotides; *ori*: origin of replication;p-value:probability; PCR: polymerase chain reaction; P_TK_: thymidine kinase promoter; Qpcr: quantitative real-time polymerase chain reaction; r: BestKeeper coefficient of correlation; *Rluc*: *Renilla *luciferase gene; *RPL13A*: ribosomal protein L13a gene; SD: standard deviation; *SDHA*: succinate dehydrogenase complex, subunit A gene; SV40: simian virus 40; TK: thymidine kinase; *TOP1*: DNA topoisomerase I gene; TPA: phorbol-12-myristate 13-acetate; *YWHAZ*: tyrosine-3-monooxygenase/tryptophan-5-monooxygenase activation protein (zeta polypeptide) gene.

## Authors' contributions

MJ, KP, PM and JY conducted the experiments. RD conducted the statistical analyses on the experimental data. MJ, WK and ARP drafted the manuscript. WK and ARP designed the project and secured funding for the study. All authors read and approved the final text.
